# Celastrol Efficacy by Oral Administration in the Adjuvant-Induced Arthritis Model

**DOI:** 10.3389/fmed.2020.00455

**Published:** 2020-09-08

**Authors:** Rita Cascão, Bruno Vidal, Tânia Carvalho, Inês Pascoal Lopes, Vasco C. Romão, João Goncalves, Luis Ferreira Moita, João Eurico Fonseca

**Affiliations:** ^1^Unidade de Investigação em Reumatologia, Faculdade de Medicina, Instituto de Medicina Molecular-João Lobo Antunes, Centro Académico de Medicina de Lisboa, Universidade de Lisboa, Lisbon, Portugal; ^2^Comparative Pathology Unit, Instituto de Medicina Molecular-João Lobo Antunes, Faculdade de Medicina, Universidade de Lisboa, Lisbon, Portugal; ^3^Serviço de Reumatologia e Doenças Ósseas Metabólicas, Hospital de Santa Maria, Centro Hospitalar Universitário Lisboa Norte, Lisbon, Portugal; ^4^Faculdade de Farmácia, iMed – Research Institute of Medicines, Universidade de Lisboa, Lisbon, Portugal; ^5^Innate Immunity and Inflammation Laboratory, Instituto Gulbenkian de Ciência, Oeiras, Portugal

**Keywords:** rheumatoid arthritis, adjuvant-induced arthritis, celastrol, dose, efficacy

## Abstract

**Background:** We previously demonstrated that celastrol has significant anti-inflammatory and bone protective effects when administered via the intraperitoneal route. For further preclinical evaluation, an effective oral administration of celastrol is crucial. Here we aimed to study the therapeutic dose range for its oral administration.

**Methods:** Celastrol (1–25 μg/g/day, *N* = 5/group) was administrated orally to female adjuvant-induced arthritis (AIA) rats after 8 days of disease induction for a period of 14 days. A group of healthy (*N* = 8) and arthritic (*N* = 15) gender- and age-matched Wistar rats was used as controls. During the treatment period, the inflammatory score, ankle perimeter, and body weight were measured. At the end of the treatment, the animals were sacrificed, blood was collected for clinical pathology, necropsy was performed with collection of internal organs for histopathological analysis, and paw samples were used for disease scoring.

**Results:** Doses higher than 2.5 μg/g/day of celastrol reduced the inflammatory score and ankle swelling, preserved joint structure, halted bone destruction, and diminished the number of synovial CD68+ macrophages. Bone resorption and turnover were also reduced at 5 and 7.5 μg/g/day doses. However, the dose of 7.5 μg/g/day was associated with thymic and liver lesions, and higher doses showed severe toxicity.

**Conclusion:** Oral administration of celastrol above 2.5 μg/g/day ameliorates arthritis. This data supports and gives relevant information for the development of a preclinical test of celastrol in the setting of a chronic model of arthritis since rheumatoid arthritis is a long-term disease.

## Introduction

The therapeutic effect of celastrol has been demonstrated in several inflammatory diseases. Celastrol is a pentacyclic-triterpene compound that can be found in root extracts from *Tripterygium wilfordii* (TW) (1), an herb used in Chinese medicine (2–4).

In the last 5 years, increasing evidence for the therapeutic potential of celastrol in the treatment of rheumatoid arthritis (RA) has emerged. Studies have suggested that the anti-inflammatory properties of celastrol can be mainly attributed to the regulation of cytokine production (5–8), the modulation of inflammatory cells (8–13), the inhibition of osteoclastogenesis, and bone protection (8, 14–16), mostly due to its ability to downregulate the NF-kB pathway.

Specifically, we have demonstrated that the intraperitoneal administration of celastrol suppressed inflammatory signs (7), preserved joint structure, with abrogation of the inflammatory infiltrate and cellular proliferation (7, 8), and halted focal bone damage in the adjuvant-induced arthritis (AIA) rat model (8, 17). This inhibitory effect of cellular infiltration and proliferation may prove to be of interest to treat the development of the synovial tumor-like pannus tissue characteristic of RA, one of the main contributors to bone damage. Importantly, we have reported that this compound is able to significantly decrease the number of sublining CD68+ synovial macrophages (8), a biomarker of treatment efficacy in RA (18–20). So far, we observed that the intraperitoneal administration of celastrol to AIA rats was not associated with overt signs of toxicity (8).

Despite the therapeutic potential of celastrol, further clinical application seems to be limited by poor water solubility (21), low oral bioavailability (22), possible side effects (23–26), and variability in dose regimens (9, 27–30).

Therefore, in order to advance the preclinical development of celastrol as a candidate therapeutic compound for RA treatment, we analyzed the therapeutic dose range for oral administration of pure celastrol using the AIA rat model.

## Materials and Methods

### Animals

The AIA model has been the most extensively used arthritic rat model to study anti-arthritic agents because it has an excellent track record for predicting both activity and toxicity. AIA rats share key features related to RA, making them a critical tool in drug development, and exhibit the greatest magnitude of disease when compared with other models of arthritis (31). Eight-week-old female Wistar AIA rats weighing 230–250 g were purchased from Charles River Laboratories International (Massachusetts, USA). Charles River Laboratories performed the induction of adjuvant disease using Freud's complete adjuvant, supplemented with mycobacterium, and injected in the right footpad. The AIA rats were maintained under specific-pathogen-free conditions, randomly housed per group under standard laboratory conditions (at 22°C under 10-h light/14-h dark conditions), and given free access to food (RM3, SDS Diets, UK) and water (ultrapure). In addition, to minimize animal discomfort, paper shavings were used as bedding material in Double Decker GR1800 cages (Techniplast, UK) with five animals each. The criteria for a humane sacrifice were determined as previously published (8), and the animals were sacrificed when presenting the maximum inflammatory score in more than two limbs or when weight loss exceeded 20%. In accordance with Directive 2010/63/EU, all animal procedures were approved by the institutional animal welfare body (ORBEA-iMM) and licensed by the Portuguese competent authority (DGAV—Direcção Geral de Alimentação e Veterinária, license number: 0421/000/000/2016).

### Celastrol Preparation and Administration

Celastrol (Sigma, Missouri, USA) stock solution of 10 mg/ml was prepared using ethanol 100% as solvent (vehicle). A recent study has tested the solubility of celastrol in different vehicles, and we have previously demonstrated that ethanol is one of the most efficient solvents for this compound (21). This celastrol stock solution was further dissolved in PEG400 (Sigma, St. Louis, USA) (1, 2.5, 5, 7.5, 12.5, and 25 μg/g in 1 ml) and administrated by oral gavage to AIA rats for 14 consecutive days (*N* = 5 rats/group). The sample size in each group was calculated using free sample size calculating G^*^Power version 3.1.9.2 software [type of power analysis: *a priori*; α error probability: 0.05; power (1-β error probability): 0.95; effect size d: 2.59; actual power: 0.976]. This calculation was based on our own previous data (7, 8, 17).

Our study was approved by the institutional animal welfare body, licensed by the Portuguese competent authority, and complied with good ethical, scientific, legal, and economic reasons for using laboratory animals, including the 3R principle (replace, reduce, and refine). Focused on the “reduce” rule, we used the minimum number of animals, calculated by the free sample size calculating G^*^Power software, in order to perform this study.

The need for daily administrations is supported by the study of Zhang et al., which showed that the half-life of pure celastrol is ~10 h (22). Based on this publication, we have also calculated the oral dose of 2.5 μg/g/day as the equivalent to the intraperitoneal dose of 1 μg/g/day that we had previously found to be effective in the treatment of arthritis in the same rat model (7, 8). The calculation was based on the fraction of the celastrol dose absorbed in the portal blood after oral administration, which was 17.6%. Intraperitoneal dose calculation took into consideration that the intraportal dose will have higher bioavailability. Therefore, the relationship between the area under the curve and the doses administered in the oral and the intraportal dose was used for determining the intraperitoneal dose. Treatment was initiated after 8 days of disease induction, at the acute clinical stage of arthritis progression (therapeutic intervention) (32). Healthy non-arthritic (*N* = 8) and arthritic untreated (*N* = 15) female age-matched Wistar rats were used as controls. The arthritic untreated group received an equal volume of vehicle through oral gavage. The vehicle proportion of ethanol and PEG400 used was in the same proportion as the one used in the celastrol-treated groups.

The rats were sacrificed after 22 days of disease progression by CO_2_ narcosis, and blood, internal organs, as well as paw samples were collected. Studies using the AIA model are generally completed at this time point due to a plateau effect of inflammatory manifestations (7, 31).

### Arthritis Severity Evaluation

Disease activity was clinically evaluated during the period of treatment by two independent investigators using an inflammatory score and by measuring the ankle perimeter as readout of articular swelling. The inflammatory score was measured by counting the score of each limb joint in a scale of 0–3 (0—absence, 1—erythema, 2—erythema and swelling, and 3—deformities and functional impairment). The total score for each animal was defined as the sum of the partial scores of each affected joint (7, 33). Body weight was also registered, every 2 days, throughout the experimental procedure.

### Clinical Pathology and Histological Analysis

Blood was collected from the heart and used for serum biochemistry measurement of creatine kinase (CK), urea, lactate dehydrogenase (LDH), alanine transaminase (ALT) (BioAssay Systems, California, USA), and pro-ANP (Biomedica, Viena, Austria) by enzyme-linked immunosorbent assay (ELISA) technique, according to the manufacturer's instructions. The ELISA measurement was performed using the plate reader Infinite M200 (Tecan, Mannedorf, Switzerland).

Necropsy was performed, and the left hind paw was collected for tibiotarsal joint histopathological analysis and disease scoring, and the liver, spleen, kidney, lung, thymus, heart, gastrointestinal tract, and long bone (humerus) were collected for routine histopathological analysis to assess signs of celastrol-induced toxicity. Briefly, all organs and tissues were immediately fixed in 10% neutral buffered formalin, the bones were further decalcified in 10% formic acid, and all samples were processed for paraffin embedding.

For histological disease activity scoring, serial 5-μm sections of the tibiotarsal joints were stained with hematoxylin and eosin (H&E) and immunohistochemistry was performed using the following antibodies: mouse monoclonal anti-CD68 (Abcam, Cambridge, UK), mouse monoclonal anti-osteocalcin (osteoblast marker; indicator of osteoblastic activity; Abcam, Cambridge, UK), rabbit polyclonal anti-cathepsin K (osteoclast marker; mature osteoclast enzyme; Biorbyt, Cambridge, UK), and rabbit polyclonal anti-Ki67 (Abcam, Cambridge, UK) antibodies. The tissue sections were incubated with the primary antibody and with EnVision+ (Dako, Glostrup, Denmark). Color was developed in a solution containing diaminobenzadine-tetrahydrochloride (Sigma, Missouri, USA) and 0.5% H_2_O_2_ in phosphate-buffered saline buffer (pH 7.6). The slides were counterstained with hematoxylin and mounted. Histological disease activity scoring in the tibiotarsal joints was performed by an independent researcher blinded to the experimental groups using four semi-quantitative scores, as previously published: sublining layer infiltration score (0—none to diffuse infiltration, 1—lymphoid cell aggregate, 2—lymphoid follicles, and 3—lymphoid follicles with germinal center formation), lining layer cell number score (0—fewer than three layers, 1—three to four layers, 2—five to six layers, and 3—more than six layers), bone erosion score (0—no erosions, 1—minimal, 2—mild, 3—moderate, and 4—marked), and global disease severity score (0—no signs of inflammation, 1—mild, 2—moderate, and 3—severe) (7, 34, 35). The proliferation of synoviocytes was also analyzed using a semi-quantitative score (0–4) of Ki67 immunostaining (0—no stained cells, 1−0–25% staining, 2−25–50% staining, 3−50–75% staining, and 4—more than 75% stained cells) (7). Images were acquired in a Leica DM2500 (Leica Microsystems, Wetzlar, Germany) coupled to a Leica MC170 HD microscope camera.

For the assessment of celastrol-induced toxicity, 4-μm sections of the liver, spleen, kidney, lung, thymus, heart, gastrointestinal tract, and humerus were stained with H&E and analyzed by a pathologist (TC) blinded to the experimental groups. The slides were scanned and images were acquired by a Hamamatsu NanoZoomerSQ slide scanner. The classification of lesions followed previously published criteria (36–42).

### Measurement of Serum Bone Turnover and Resorption Markers

Bone turnover was analyzed by quantifying the levels of tartrate-resistant acid phosphatase 5b (TRACP-5b), procollagen type 1 amino-terminal propeptide (P1NP), and C-terminal cross-linked telopeptide of type I collagen (CTX-I) in serum using ELISA (Immunodiagnostic System, Boldon, UK). All commercial assays were performed according to the manufacturers' instructions, and standard curves were generated using the supplied reference concentrations. Measurement was performed using a plate reader Infinite M200 (Tecan, Mannedorf, Switzerland).

### Statistical Analysis

Normality distribution was assessed by D'Agostino and Pearson test. The treated groups (celastrol 1, 2.5, 5, and 7.5 ug/g) were compared against the untreated arthritic group with the Mann–Whitney test with Bonferroni correction to account for multiple comparisons, as previously reported (17). Thus, applying the Bonferroni correction, we divided the global significance level at 0.05 by the number of independent tests (*n* = 4) to get the Bonferroni critical value of *p* < 0.0125, below which a test would be significant. In addition, the Mann–Whitney test was also used to compare differences between the other two independent groups: healthy non-arthritic and untreated arthritic rats. For paired samples (e.g., different time points), we have used the Wilcoxon matched-pairs signed-rank test. In these cases, *p* < 0.05 were considered to be significant. All statistical analyses were performed using the GraphPad Prism V.5.01 (GraphPad, California, USA). Data were presented as median with interquartile range.

## Results

### Oral Celastrol Improved the Clinical Outcome and Ameliorated the Histopathological Aspects of AIA Rats

**Figure 1:**
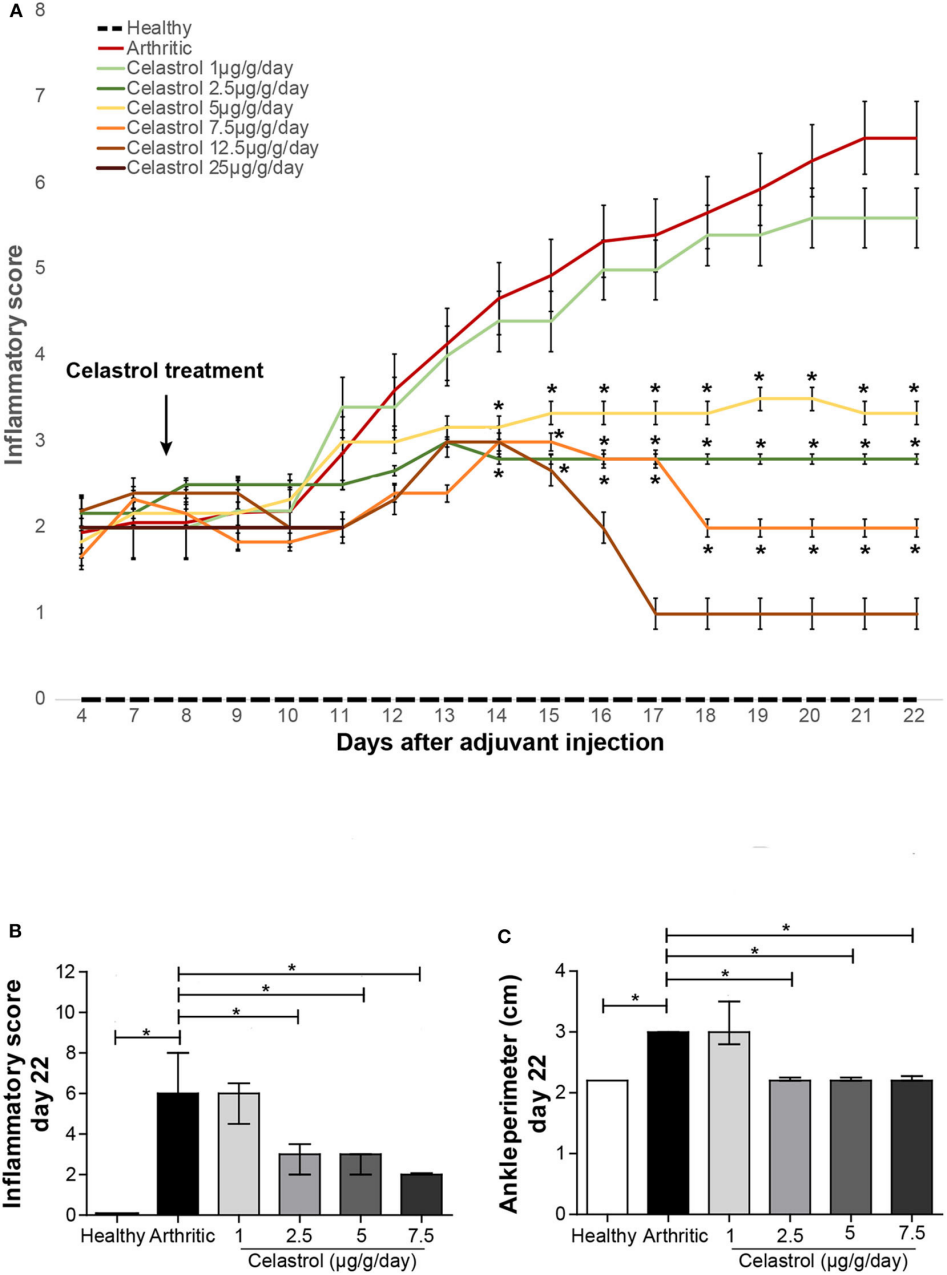
Oral celastrol significantly improved the clinical outcome in adjuvant-induced arthritis rats. The inflammatory score measured throughout the treatment period **(A)**, the inflammatory score **(B)**, and the ankle perimeter **(C)** evaluated by day 22 after disease induction showed that the range of dosage between 2.5 and 7.5 μg/g/day was effective in preventing arthritis progression. Healthy *N* = 8, arthritic *N* = 17, celastrol 1 μg/g/day *N* = 5, celastrol 2.5 μg/g/day *N* = 5, celastrol 5 μg/g/day *N* = 5, celastrol 7.5 μg/g/day *N* = 5, celastrol 12.5 μg/g/day *N* = 5, and celastrol 25 μg/g/day *N* = 5. The data are shown as median with interquartile range. The differences were considered as statistically significant for *p* < 0.05, according to Mann–Whitney tests for comparisons between healthy and arthritic groups and *p* < 0.0125 according to Mann–Whitney tests with Bonferroni correction for comparisons between arthritic and celastrol-treated groups. *p < 0.05 and *p* < 0.0125.

The onset of arthritis in the contralateral ankle joint to the injection site occurred approximately at day 8 post-disease induction. As demonstrated in Figure 1A, all animals showed clinical signs of arthritis by the 4th day of disease induction, and after 10 days, the untreated arthritic group showed accelerated disease progression. In contrast, after 6 and 7 days of treatment, the rats treated with 2.5 and 5 μg/g/day (*p* = 0.0111 and *p* = 0.006) and 7.5 μg/g/day(*p* = 0.010) of celastrol showed a significant lower inflammatory score compared to untreated arthritic rats. Of note, oral celastrol at 1 μg/g/day had no effect in arthritis progression. After 3 days of treatment, all animals (5 out of 5) treated with a dose of 25 μg/g/day and 3 animals (3 out of 5) treated with 12.5 μg/g/day were euthanized due to progressive weight loss (over 15% of body weight), onset of diarrhea and respiratory distress. In the group of animals treated with 7.5 μg/g/day of celastrol, only one animal (1 out of 5) was euthanized after 10 days of treatment with the same clinical signs.

After 14 days of treatment, the dosage ranging from 2.5 to 7.5 μg/g/day showed a significant anti-inflammatory effect, as assessed by the evaluation of the clinical inflammatory score (*p* = 0.0023 with 2.5 μg/g/day, *p* = 0.0016 with 5 μg/g/day, and *p* = 0.0028 with 7.5 μg/g/day vs. arthritic animals, as shown in Figure 1B) and also by the measurement of ankle perimeter (*p* = 0.0053 with 2.5 μg/g/day, *p* = 0.0053 with 5 μg/g/day and *p* = 0.0118 with 7.5 μg/g/day vs. untreated arthritic animals, as shown in Figure 1C).

Of note, no differences were observed in body weight after 14 days of treatment, comparing each dose of celastrol (up to 7.5 μg/g/day) with the vehicle control animals (Figure S1A). Additionally, when comparing, within the same group, the first and last day of treatment, we were able to observe an increase in body weight in healthy rats (*p* = 0.0078, Figure S1B) and a significant weight loss in the untreated arthritic group, as expected (*p* = 0.0074, Figure S1C). However, no weight loss was observed in AIA animals treated with celastrol using doses up to 7.5 μg/g/day (Figures S1D–G).

**Figure 2:**
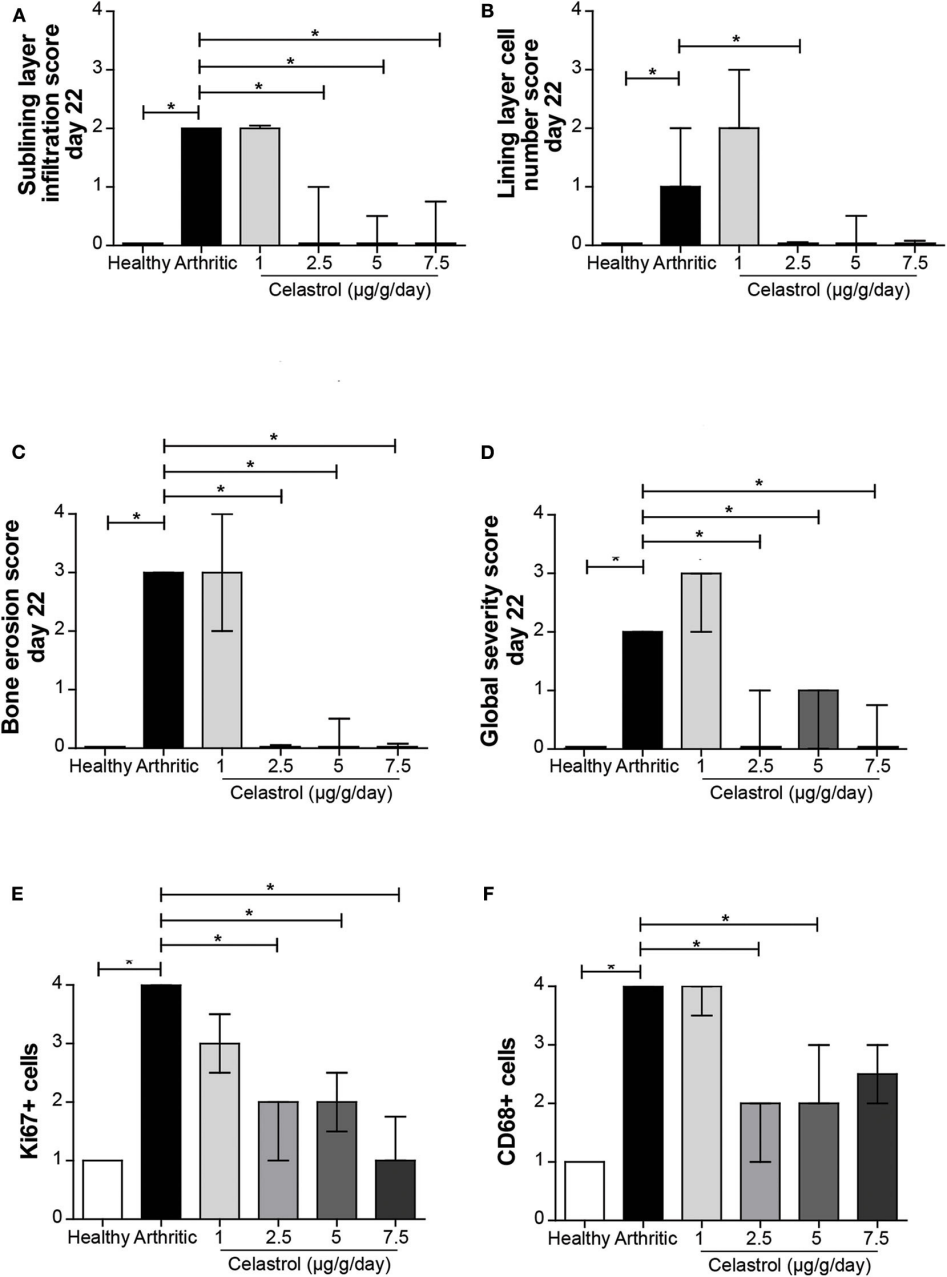
Oral celastrol treatment ameliorated the histopathological aspects of adjuvant-induced arthritis (AIA) rats and reduced synovial CD68+ macrophages. Celastrol significantly impaired inflammatory cell infiltration **(A)**, was associated with a number of synovial lining layers similar to normal values **(B)**, and reduced bone erosions **(C)**, thus preserving the normal joint structure **(D)**. The celastrol-treated AIA rats showed a significant reduction in synovial cell proliferation as assessed by the Ki67 marker **(E)** and in the number of synovial CD68-positive cells **(F)**. Healthy *N* = 8, arthritic *N* = 17, celastrol 1 μg/g/day *N* = 5, celastrol 2.5 μg/g/day *N* = 5, celastrol 5 μg/g/day *N* = 5, and celastrol 7.5 μg/g/day *N* = 4. The data are expressed as median with interquartile range. The differences were considered as statistically significant for *p* < 0.05, according to Mann–Whitney tests for comparisons between healthy and arthritic groups and *p* < 0.0125 according to Mann–Whitney tests with Bonferroni correction for comparisons between arthritic and celastrol-treated groups. *p < 0.05 and *p* < 0.0125.

As shown in Figure 2, synovial hyperplasia and marked inflammatory cell infiltration were seen in tibiotarsal joints of untreated arthritic rats (*p* = 0.0019 and *p* = 0.0001 vs. healthy controls, respectively), also associated with significant bone erosion (*p* = 0.0008 vs. healthy controls). In contrast, in celastrol-treated rats, inflammatory infiltrates were reduced in all dose regimens, except for the 1 μg/g/day group (Figure 2A, *p* = 0.0010 in 2.5 μg/g/day, *p* = 0.0006 in 5 μg/g/day and *p* = 0.0016 in 7.5 μg/g/day vs. arthritic rats). We also observed a reduction in the number of cells present in the synovial lining layer with the dose of 2.5 μg/g/day (Figure 2B, *p* = 0.0110 vs. arthritic rats), and a marked tendency to decrease in the 5 and 7.5 μg/g/day doses (*p* = 0.0220; *p* = 0.0225, respectively, vs. arthritic rats). These concentrations of celastrol were also effective in preventing bone articular destruction (Figure 2C, *p* = 0.0053 in 2.5 μg/g/day, *p* = 0.0069 in 5 μg/g/day, and *p* = 0.0118 in 7.5 μg/g/day vs. arthritic rats), with animals presenting a normal joint structure at the end of the study period (Figure 2D, *p* = 0.0028 in 2.5 μg/g/day, *p* = 0.0048 in 5 μg/g/day, and *p* = 0.0041 in 7.5 μg/g/day vs. arthritic rats).

In addition, we observed that animals treated with celastrol in the dose range between 2.5 and 7.5 μg/g/day have reduced synovial cell proliferation, as assessed in the tibiotarsal joints by Ki67 immunostaining (*p* = 0.0020, *p* = 0.0055, and *p* = 0.0026 vs. arthritic animals, respectively) (Figure 2E).

Finally, we observed that untreated arthritic rats had a higher number of infiltrating CD68+ synovial macrophages as compared to healthy controls (*p* = 0.0001) (Figure 2F). Celastrol administration was associated with a significant decrease in the number of CD68+ macrophages infiltrating the arthritic joint (*p* = 0.0014 in 2.5 μg/g/day, *p* = 0.0095 in 5 μg/g/day vs. arthritic rats; for the 7.5 μg/g/day there is a strong tendency although not reaching significance, *p* = 0.0213).

### Oral Celastrol Reduced the Number of Joint Osteoclasts and Osteoblasts

**Figure 3:**
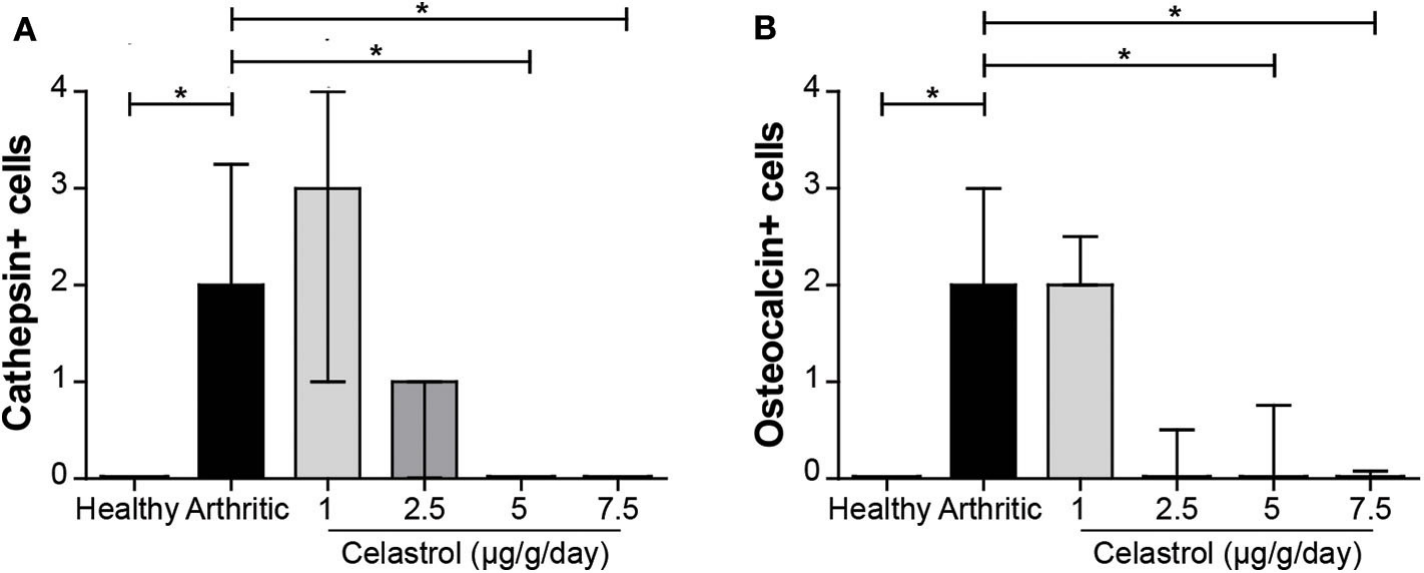
Oral celastrol reduced the number of osteoclasts and osteoblasts in arthritic joints. Cathepsin k-positive cells **(A)** and osteocalcin-positive cells **(B)** were identified in arthritic joints by immunohistochemistry. Celastrol treatment significantly reduces both types of cells in the doses of 5 and 7.5 μg/g/day. The paw samples were collected at the time of sacrifice. Healthy *N* = 8, arthritic *N* = 17, celastrol 1 μg/g/day *N* = 5, celastrol 2.5 μg/g/day *N* = 5, celastrol 5 μg/g/day *N* = 5, and celastrol 7.5 μg/g/day *N* = 4. The data are expressed as median score with interquartile range. The differences were considered as statistically significant for *p* < 0.05, according to the Wilcoxon signed-rank tests for comparisons between healthy and arthritic groups and *p* < 0.0125 according to Mann–Whitney and Wilcoxon signed-rank tests with Bonferroni correction for comparisons between arthritic and celastrol-treated groups. *p < 0.05 and *p* < 0.0125.

The immunolocalization of osteoclasts and osteoblasts in subchondral bone tissue at the tibia/talus region was performed to evaluate a possible celastrol dose-dependent effect on bone remodeling. Untreated arthritic rats showed increased osteoclast numbers (cathepsin k+ cells) in the tibiotarsal bones (*p* = 0.004 vs. healthy controls, Figure 3A). Importantly, celastrol administration was associated with a significant decrease in the number of osteoclasts, to levels similar to healthy controls (*p* = 0.004 in 5 μg/g/day and *p* = 0.004 in 7.5 μg/g/day vs. arthritic rats). Untreated arthritic rats also showed increased numbers of osteoblasts (osteocalcin-positive cells) (*p* = 0.0005 vs. healthy controls, Figure 3B), a phenotype also reversed by celastrol administration, with a significant reduction in the number of osteoblasts as compared to AIA rats (*p* = 0.0005 in 5 μg/g/day and *p* = 0.0005 in 7.5 μg/g/day) reaching levels similar to healthy controls. In the case of rats treated with celastrol at the dose of 2.5 μg/g/day there is a tendency toward a decrease in the number of osteoblasts that did not reach statistical significance (*p* = 0.02 vs. untreated AIA rats).

### Oral Celastrol Reduced Bone Turnover and Resorption Markers in AIA Rats

**Figure 4:**
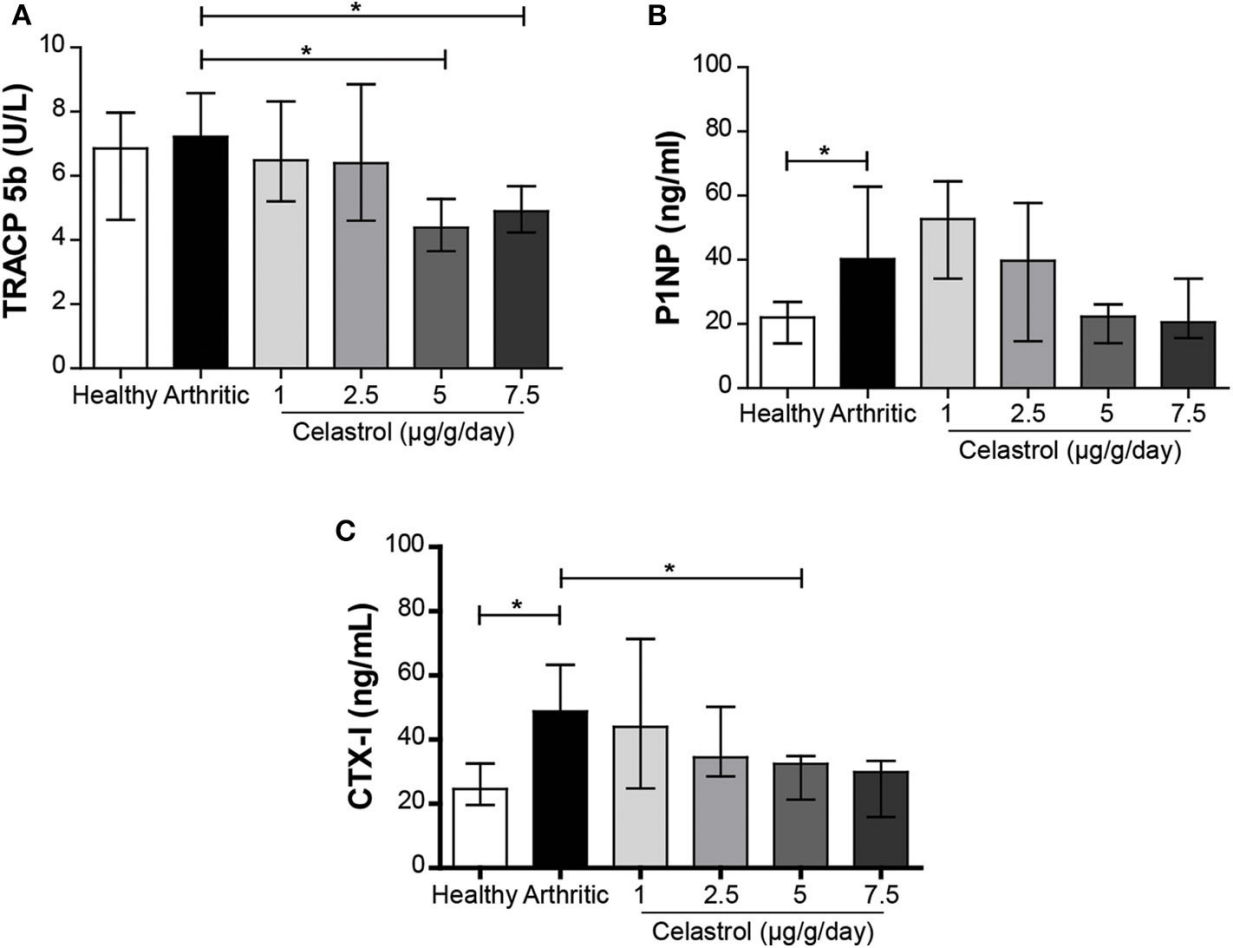
Oral celastrol was associated with the reduction of bone turnover and resorption markers. TRACP-5b **(A)**, P1NP **(B)**, and CTX-I **(C)** levels were quantified in rat serum samples collected at the time of sacrifice. Celastrol seems to reduce the levels of TRACP-5b, P1NP, and CTX-I in the treated animals in comparison with the arthritic rats. Healthy *N* = 8, arthritic *N* = 17, celastrol 1 μg/g/day *N* = 5, celastrol 2.5 μg/g/day *N* = 5, celastrol 5 μg/g/day *N* = 5, and celastrol 7.5 μg/g/day *N* = 4. The data are expressed as median with interquartile range. The differences were considered as statistically significant for *p* < 0.05, according to Mann–Whitney tests for comparisons between healthy and arthritic groups and *p* < 0.0125 according to Mann–Whitney tests with Bonferroni correction for comparisons between arthritic and celastrol-treated groups. *p < 0.05 and *p* < 0.0125.

In untreated arthritic rats, there was no significant increase in TRACP-5b levels at the end of the study period (Figure 4A). Importantly, both 5 and 7.5 μg/g/day doses of celastrol reduced TRACP-5b levels, when compared with untreated arthritic rats (*p* = 0.0031 and *p* = 0.0065, respectively), suggesting a decrease in bone resorption. In addition, the elevated levels of P1NP observed in untreated arthritic rats in comparison with healthy controls (*p* = 0.0074, Figure 4B) presented a marked tendency to decrease with the celastrol dose of 5 μg/g/day (*p* = 0.0145 vs. untreated arthritic animals). In accordance, celastrol administration was able to significantly reduce the CTX-I levels (*p* = 0.0079 for 5 μg/g/day, Figure 4C) and also induced a strong tendency to decrease with the 7.5 μg/g/day dose, (*p* = 0.0159) when compared with untreated arthritic rats, which showed an accelerated bone turnover with high levels of CTX-I (*p* = 0.0016 vs. healthy controls). Also, the CTX-I serum levels had a tendency toward a decrease at 2.5 μg/g/day, but it did not reach statistical significance.

### Higher Dose of Celastrol Was Associated With Signs of Toxicity

A histopathological analysis of selected organs showed histological changes associated with the disease model and histological changes associated with the test compound.

Concerning the disease-related histopathological aspects, giant-cell granulomas were seen in liver, lung, and spleen in all groups, with varied severity unrelated with treatment or dose levels (Figure S2). Grossly, multiple gray to gray-brown foci were observed over the pleural surface of the lung and the surface of the liver and spleen. Microscopically, these changes corresponded to the variably sized multifocal to coalescing granulomas, characterized by dense aggregates of mostly macrophages admixed with frequent multinucleated giant cells and fewer lymphoid cells (Figure S3). In the liver, these granulomas were present in the parenchyma; in the lung, they were mostly arranged around bronchioles, around or adjacent to large vessels; and in the lung interstitium and in the spleen they were mostly seen in the red pulp.

Regarding possible celastrol-induced histopathological aspects, lesions were seen exclusively in the thymus and the liver of medium- to high-dose groups, and the severity grade was related with dose (Figure S4). The thymus showed lymphocyte necrosis, multifocal, and mild to moderate depletion of lymphocytes in the cortical zone. Severity ranged from moderate to marked and was seen exclusively in two of six animals of the high-dose group (7.5 μg/g/day) (Table 1). In the liver, mild lymphocyte-rich peribiliary inflammatory cell infiltration, and bile duct hyperplasia were only seen in two and three out of five animals, respectively, in the 2.5 μg/g/day dose-level group (Table 1). Higher toxicity incidence was observed in the 5 and 7.5 μg/g/day dose groups, with a toxicity severity grade ranging from mild to moderate in the medium dose and from mild to marked in the high dose. Due to the design of the experiment and to the use of an acute rat model of arthritis, which rapidly progresses and resolves, it was not possible to assess if changes associated with celastrol treatment were reversible as no analysis was done after suspension of the compound. No significant histological changes were seen in any other organs.

**Table 1:** Histopathological findings in Wistar rats upon celastrol treatment at different dose levels.

**Target organ**	**Effect**	**Severity grade^**a**^**	**Celastrol (μg/g/day)**				
			**0**	**1**	**2.5**	**5**	**7.5**
Thymus	Necrosis, lymphocyte	(+2)	0/16	0/5	0/5	0/6	1/6
		(+3)	0/16	0/5	0/5	0/6	1/6
Liver	Inflammatory cell infiltration, peribiliary (intrahepatic)	(+1)	0/16	0/5	2/5	5/6	2/6
		(+2)	0/16	0/5	0/5	0/6	2/6
		(+3)	0/16	0/5	0/5	0/6	2/6
	Bile duct hyperplasia	(+1)	0/16	0/5	3/5	4/6	4/6
		(+2)	0/16	0/5	0/5	1/6	2/6

a *Severity grade: (+1), mild; (+2), moderate; (+3), marked*.

**Figure 5:**
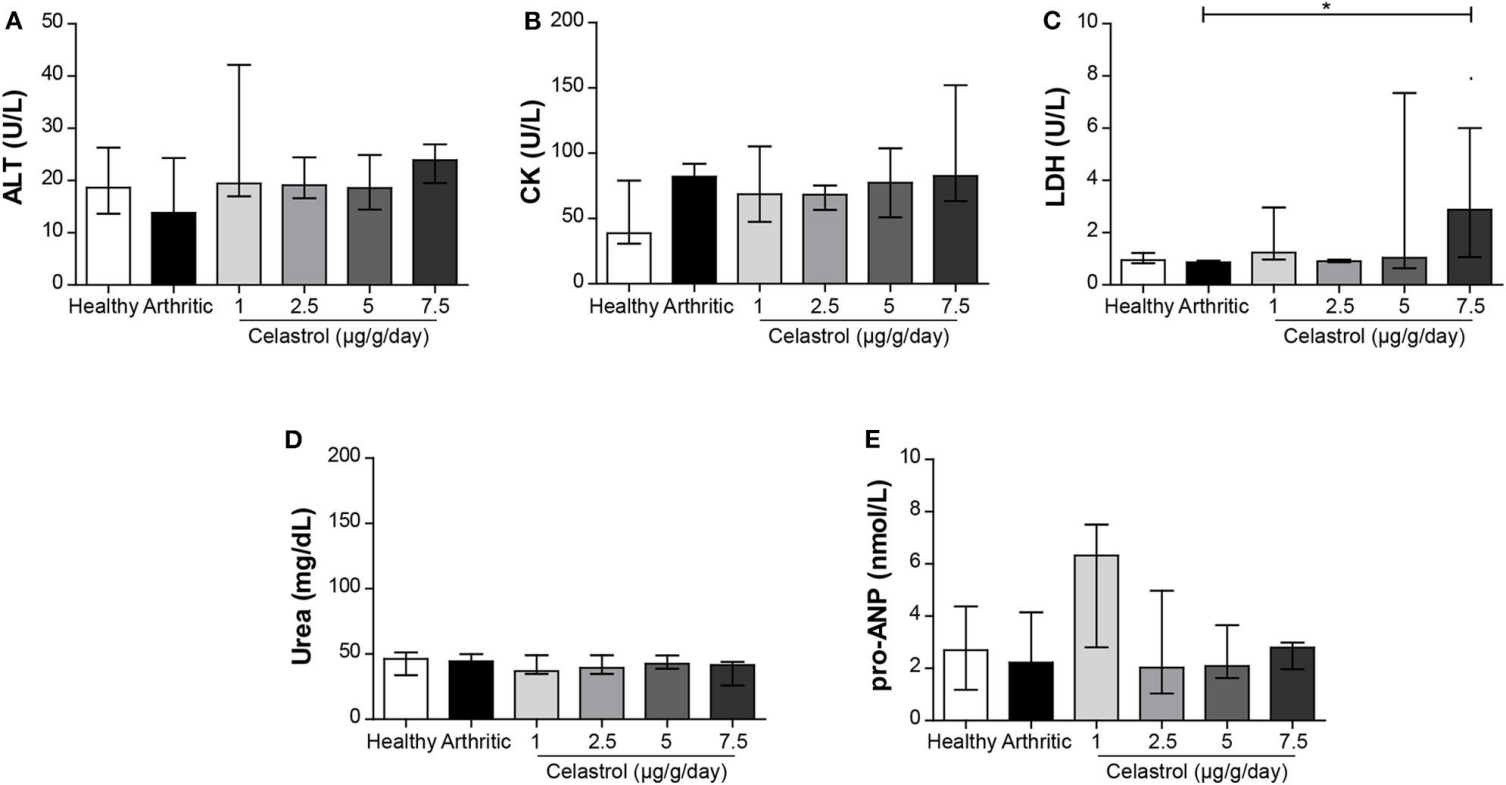
Serum biochemistry for liver, renal, and cardiac markers upon oral administration of celastrol. The serum levels of ALT **(A)**, CK **(B)**, LDH **(C)**, urea **(D)**, and pro-ANP **(E)** were measured by ELISA to evaluate liver, kidney, and cardiac toxicity. Only the levels of LDH were increased in animals treated with 7.5 μg/g/day of celastrol. Healthy *N* = 8, arthritic *N* = 10, celastrol 1 μg/g/day *N* = 5, celastrol 2.5 μg/g/day *N* = 5, celastrol 5 μg/g/day *N* = 5, and celastrol 7.5 μg/g/day *N* = 4. The data are expressed as median with interquartile range. The differences were considered as statistically significant for *p* < 0.05, according to Mann–Whitney tests for comparisons between healthy and arthritic groups and *p* < 0.0125 according to Mann–Whitney tests with Bonferroni correction for comparisons between arthritic and celastrol-treated groups. *p < 0.05 and *p* < 0.0125.

Moreover, the serum biochemistry for ALT, urea, pro-ANP, and CK showed levels within the reference range of the laboratory for this strain/species (Figures 5A,B,D,E). LDH was increased at the dosage level of 7.5 μg/g/day (as shown in Figure 5C, *p* = 0.0062 vs. untreated arthritic rats, respectively). The electrocardiogram remained unaltered in celastrol-treated animals (data not shown).

## Discussion

In this study, we aimed to determine the therapeutic oral dose of pure celastrol and the associated toxicity signs in the AIA rat model.

The doses of celastrol were extrapolated from the intraperitoneal dose used in our previous studies (7, 8, 17), also taking into consideration the bioavailability and the pharmacokinetics of pure celastrol upon oral administration in rats (22).

We observed that, after 3 days of treatment, all animals treated with 25 μg/g/day of celastrol and more than 50% of those treated with 12.5 μg/g/day showed a significant decline in health status, with progressive weight loss, diarrhea, and respiratory distress, all of these unrelated with the disease model, and were euthanized. These data suggest that the dose of 25 μg/g/day is the lethal dose (LD) and the dose of 12.5 μg/g/day is the LD_50_ for the oral administration of pure celastrol, and these dose groups were therefore excluded from this study. After 10 days under celastrol treatment, one out of five rats treated with 7.5 μg/g/day also presented similar clinical signs and was euthanized.

We have observed that the oral dose of 1 μg/g/day of celastrol was not effective in the treatment of arthritis. In contrast, the oral doses of 2.5, 5, and 7.5 μg/g/day showed significant anti-inflammatory properties as assessed by the evaluation of the inflammatory score and ankle swelling. Most of the inflammatory and bone-related parameters showed a dose-dependent effect. However, in some of the analyses, this effect did not reach statistical significance, which might be due to the smaller sample size in these groups. The celastrol doses of 2.5, 5, and 7.5 μg/g/day were able to reduce synovial cell infiltration and proliferation and also decreased bone erosions in joints. Importantly, at the doses of 2.5 and 5 μg/g/day and with a strong tendency at the dose of 7.5 μg/g/day, celastrol reduced the number of sublining CD68+ synovial macrophages, a biomarker of clinical response (18–20). These data suggest that, at these oral dosages, celastrol is effective for the treatment of arthritis. Additionally, the doses of 5 and 7.5 μg/g/day reduced the number of osteoclasts and osteoblasts present in joint tissues. This observation is in agreement with the reduction of the bone resorption marker TRACP-5b and also with the decrease of the bone resorption marker CTX-I in the 5 μg/g/day treated group and a tendency to diminish also at the 7.5 μg/g/day dose, suggesting the control of the accelerated bone turnover characteristic of arthritis. Our results are in line with others, where circulating levels of TRACP-5b are not affected by arthritis induction, contrarily to the marked osteoclastic activity occurring in this arthritic model. However, there are increased levels of TRACP-5b in the protein extracts obtained from inflamed joints (43). These findings suggest that TRACP-5b reflects bone resorption more accurately when measured locally rather than systemically (43, 44). Regarding TRACP-5b, CTX-I, and P1NP results, celastrol might have a direct effect on bone metabolism in the setting of inflammation. Importantly, these results suggest that the oral administration of celastrol is also able to contribute to the prevention of bone damage, as we have previously demonstrated using AIA rats under treatment with 1 μg/g/day of celastrol via intraperitoneal route (17).

Some inflammatory disturbances were still noted in the treated animals. This is a consequence of the inflammatory process that occurred during the first days after disease induction (from day 0 up to day 8) but before celastrol treatment. This is in accordance with our previous observations showing that inflammation induces changes since the first days of arthritis development (45, 46).

Several studies have demonstrated that celastrol has cellular targets in the context of RA, such as TAK1/IKK and MAPK/MEK pathways as well as MMP-9, STAT3, RANKL, and MD2/TLR4, interfering with the production of cytokines, chemokines, and inflammatory mediators; inhibiting cell invasion and proliferation; and suppressing bone resorption (5, 9, 14, 47). In accordance, Liu et al. have observed in a mouse model of dexamethasone-induced secondary osteoporosis that celastrol not only improves lipid metabolism and reduces hypercalciuria but also mitigates articular cartilage lesions, decreases NF-kB, MMP-1, and MMP-9 expression, and reduces serum PTH, TRACP-5b, CTX-I, as well as deoxypyridinoline (48). In 2012, our group showed that celastrol decreases the secretion of both IL-1β and TNF in the THP-1 macrophage-like cell line associated not only with NF-kB inhibition but also with caspase-1 inactivation (7).

Despite its promising anti-arthritic effects, celastrol has been reported to induce weight loss in mice models of cancer (28, 49–51). At these dosages of celastrol, no body weight variations were observed, suggesting no major toxicological effects induced by celastrol or by the solvents (ethanol and PEG400) used. These two agents are the most commonly employed (52) and were identified as the adequate ones for dissolving celastrol (22). However, some studies have suggested that ethanol and PEG400 may be deleterious for the gastrointestinal tract (53, 54), constituting a risk factor for toxicological side effects such as body weight loss. Since no variations in body weight were observed in celastrol-treated rats and due to the short-term treatment duration of this study, together with the fact that we have used the minimum PEG and ethanol concentrations required in order to get celastrol solubility, we can exclude this deleterious side effect in the gastrointestinal tract. No clinical chemistry or histopathological toxicity of this compound has been previously shown. In parallel with the assessment of the therapeutic effect of celastrol at different dose levels, we also assessed its possible toxic effects at the same doses (1–7.5 μg/g/day). No changes in ALT and blood urea were detected, suggesting that it did not induce major liver or renal damage. Celastrol blocks the ion conduction of cardiac Kir2.1 and hERG potassium channels and reduces channel density on the cell surface upon chronic treatment (26), which may predict cardiotoxicity. However, the CK and the pro-ANP levels were normal and the electrocardiogram was unremarkable. LDH was increased in the lower and higher doses of celastrol. LDH is a very unspecific blood marker, and the interpretation of this result as a sign of toxicity is dubious (55).

However, the dose of 7.5 μg/g/day was associated with marked histopathologic lesions in the thymus and liver of some animals. The significance of these observations is still unclear and they have not been reported before. Model-dependent aspects cannot be excluded, and their physiologic impact on the immune system and liver is still uncertain.

In accordance with our data, similar evidences were obtained in a study that tested the efficacy and the safety of four TW preparations in collagen-induced arthritis rats (56). The authors have concluded that all plant preparations were effective in the treatment of inflammation, with no obvious hepatotoxicity or nephrotoxicity. The clinical experience with the use of TW extracts in RA patients has shown that there are improvements in symptoms and physical function. In general, the most frequent side effects reported in these clinical trials were gastrointestinal disturbances. (2, 3, 57–60) More recently, a study has shown that a combination therapy of TW extracts and methotrexate (MTX) is more effective than MTX alone (4, 61). This can be a strategy to lower the dose of TW extracts or of celastrol, thus limiting the side effects.

To more extensively evaluate the safety of orally administered celastrol, male rats could be included to provide information on gender-specific toxic effects and other animal models could explore different toxicity profiles. The acute AIA rat model of arthritis is not an adequate model to study the long-term safety and toxicity profile. Thus, the evaluation of a chronic arthritic model would be also useful to assess the effect of longer exposure and the existence of reversible side effects. Moreover, the administration of celastrol in healthy non-arthritic rats might further determine the adverse/side effects only related to the compound.

In conclusion, the results showed that 2.5 μg/g/day is the lowest effective oral dose of pure celastrol, with severe toxicity signs arising at 7.5 μg/g/day, suggesting a narrow therapeutic window. It would be now relevant to test this compound in the setting of a chronic model of arthritis using low doses in association with MTX.

## Data Availability Statement

All datasets generated for this study are included in the article/Supplementary Material.

## Ethics Statement

The animal study was reviewed and approved by Institutional animal welfare body (ORBEA-iMM) and licensed by the Portuguese competent authority (DGAV – Direcção Geral de Alimentação e Veterinária, license number: 0421/000/000/2016). In accordance with Directive 2010/63/EU all animal procedures were approved. Instituto de Medicina Molecular, Lisbon, Portugal.

## Author Contributions

RC, LM, and JF designed the study. RC conducted the study. RC, TC, IL, and BV collected the data. RC, TC, IL, BV, and VR analyzed the data. RC, TC, BV, JG, LM, and JF interpreted the data. RC and TC drafted the manuscript. RC, BV, VR, JG, LM, and JF revised the manuscript content. RC, TC, IL, VR, BV, JG, LM, and JF approved the final version of the manuscript and take responsibility for the integrity of the data analysis. All authors contributed to the article and approved the submitted version.

## Conflict of Interest

The authors declare that the research was conducted in the absence of any commercial or financial relationships that could be construed as a potential conflict of interest.
