# Supplementary material for: Celastrol Efficacy by Oral Administration in the Adjuvant-Induced Arthritis Model
Source: Front Med (Lausanne). 2020 Sep 8;7:455. doi: 10.3389/fmed.2020.00455 (PMC7505947; doi:10.3389/fmed.2020.00455)
Supplement: Supplementary file 1 [file Data_Sheet_1.docx]

**Celastrol efficacy by oral administration in the**

**adjuvant-induced arthritis model**

**SUPPLEMENTARY MATERIAL**

**
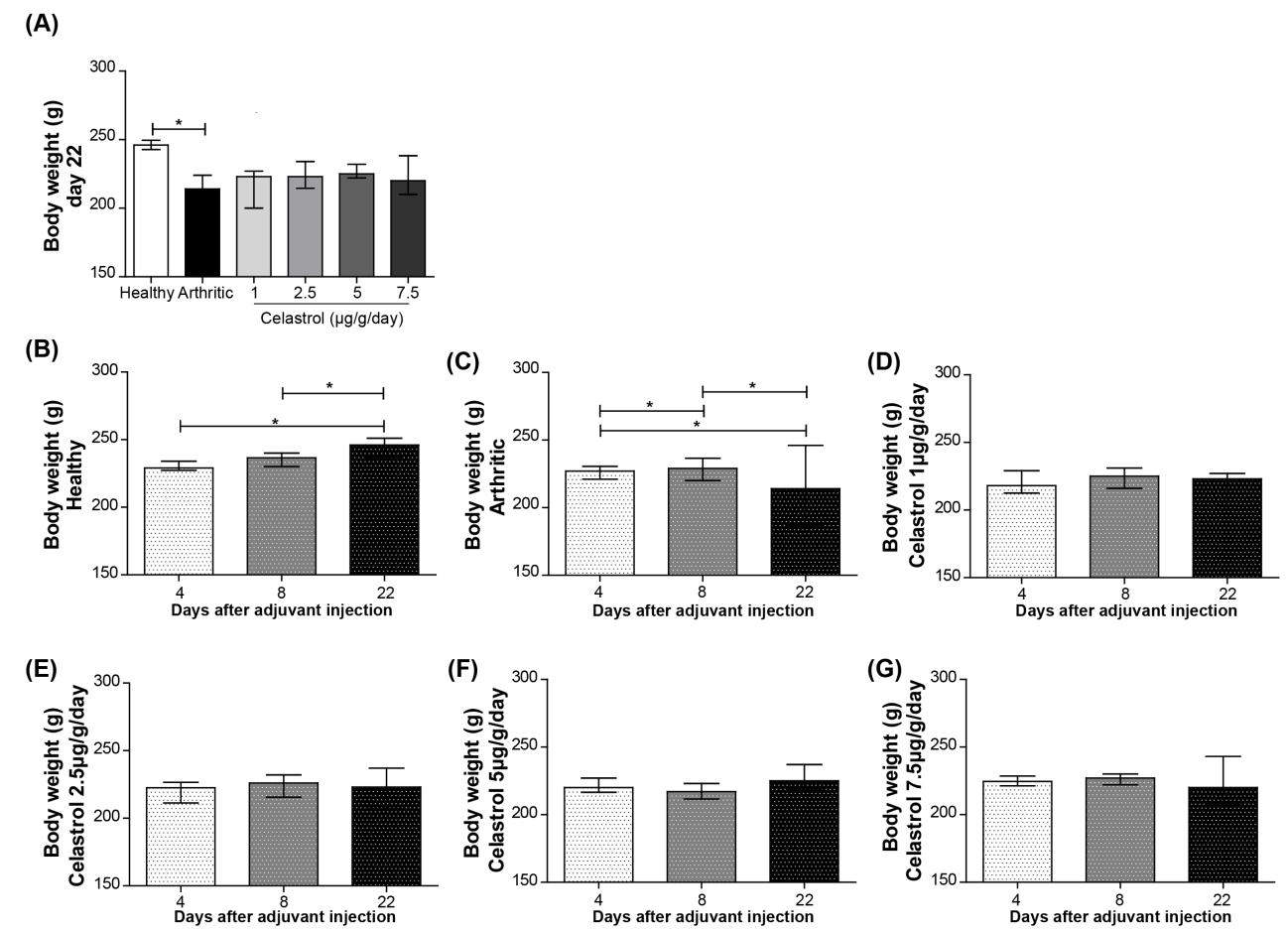
S1 Fig – Oral celastrol treatment is not associated with weight loss at doses below 7.5µg/g/day.** (A) Body weight variations throughout treatment period. (B-G) Body weight measurement at baseline (day 4) and at the first day of celastrol administration (day 8) comparing with the last day of treatment (day 22) in each group. Notice that no weight loss was observed due to celastrol administration. Contrarily, there was an association between disease severity and weight loss in arthritic rats. Healthy N=8, Arthritic N=17, Celastrol 1µg/g/day N=5, Celastrol 2.5µg/g/day N=5, Celastrol 5µg/g/day N=5 and Celastrol 7.5µg/g/day N=4. Data are expressed as median with interquartile range. Differences were considered statistically significant for p-values of p<0.05, according to the Mann–Whitney tests for figure 1A and Wilcoxon matched-pairs signed rank test for figure 1B, C, D, E, F and G.


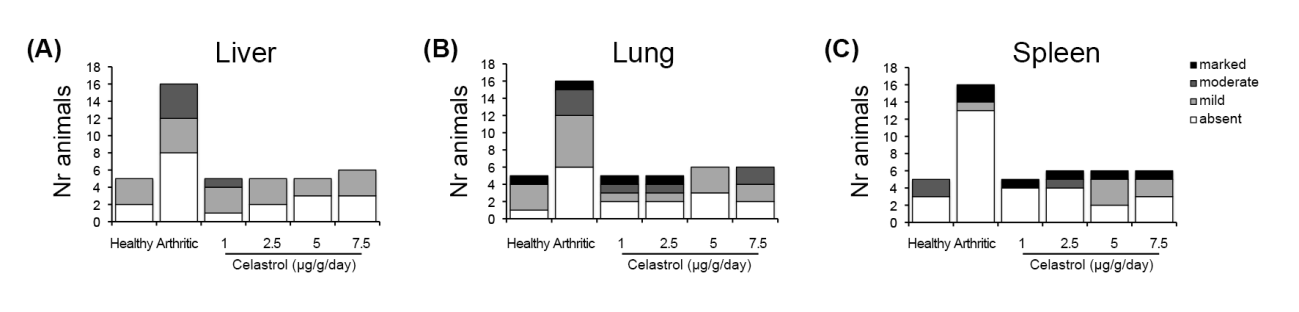


**S2 Fig – Incidence of giant-cell granulomas in liver, lung and spleen of rats.** Changes were associated with the disease (arthritis) and were not dose dependent.


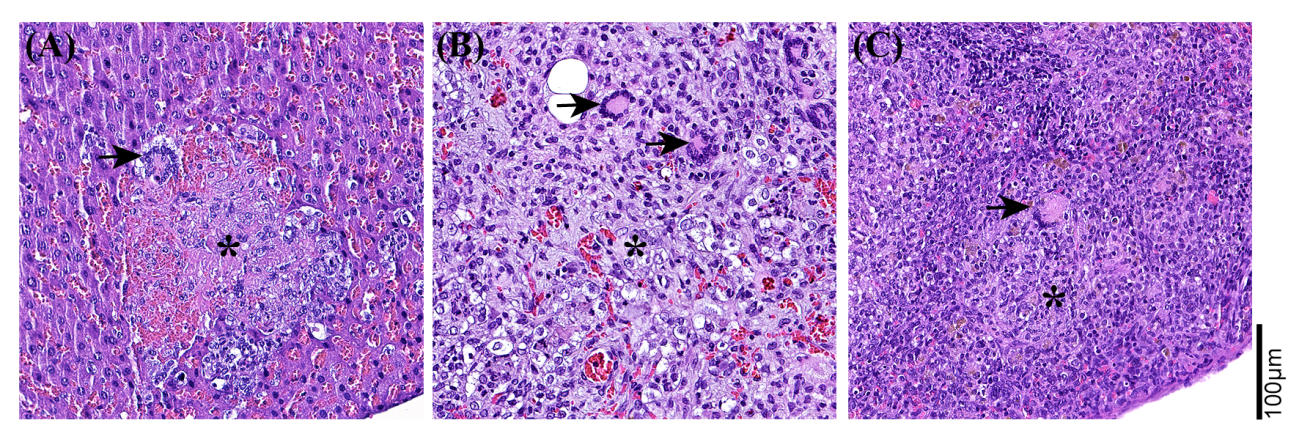


**S3 Fig – Representative microphotographs of arthritis-associated lesions in the liver, lung and spleen.** Multifocal to coalescing granulomas were seen in liver (A), lung (B) and spleen (C), and the lesions were characterized by dense aggregates mostly of macrophages (asterisk), admixed with frequent multinucleated giant cells (arrowhead), with fewer lymphoid cells. H&E, original magnification, 20x.


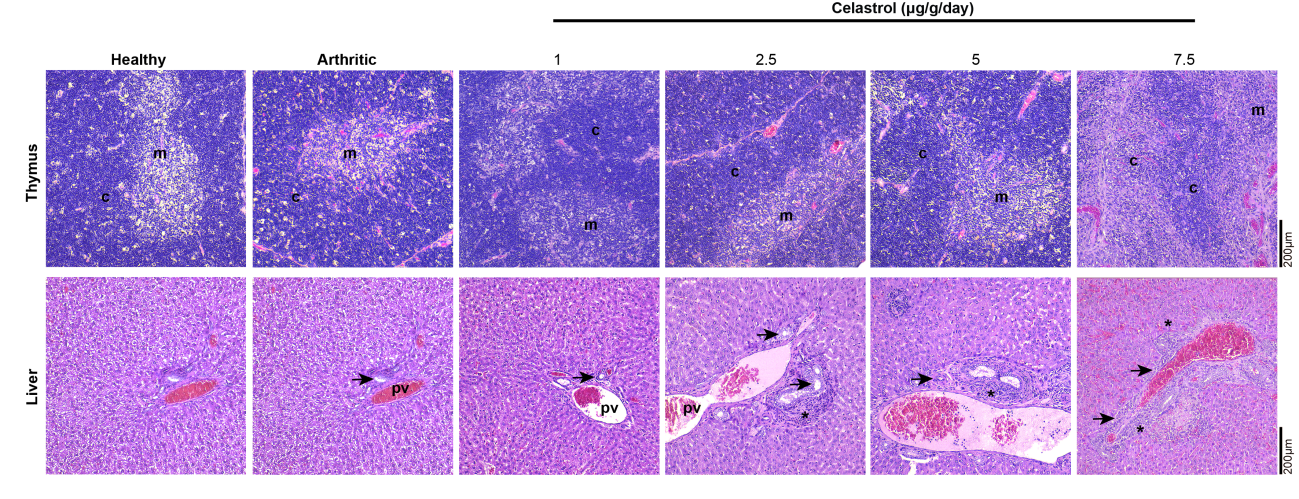


**S4 Fig – Representative microphotographs of celastrol-associated lesions in the thymus and liver.** Thymus showed moderate to severe lymphocyte necrosis (arrowhead), with loss of demarcation between cortical (c) and medullary (m) areas, exclusively in the high dose group (7.5µg/g/day). The liver shows lymphocyte-rich peribiliary inflammation (asterisk) and biliary hyperplasia (arrowhead) at the dose levels of 2.5 to 7.5µg/g/day, ranging from mild to moderate severity, the later only seen in the high dose group. H&E, original magnification, 10x.
