# Supplementary material for: Celastrol Efficacy by Oral Administration in the Adjuvant-Induced Arthritis Model
Source: Front Med (Lausanne). 2020 Sep 8;7:455. doi: 10.3389/fmed.2020.00455 (PMC7505947; doi:10.3389/fmed.2020.00455)
Supplement: Supplementary file 2 [file Table_1.docx]

**Table 1 - Histopathological findings in Wistar rats upon celastrol treatment at different dose levels.**

| **Target Organ** | **Effect** | **Severity**  **grade*** | **Celastrol (µg/g/day)** | | | | |  |
| --- | --- | --- | --- | --- | --- | --- | --- | --- |
|  |  |  | 0 | 1 | 2.5 | 5 | 7.5 | |
| Thymus | Necrosis, lymphocyte | (+2) | 0/16 | 0/5 | 0/5 | 0/6 | 1/6 | |
|  |  | (+3) | 0/16 | 0/5 | 0/5 | 0/6 | 1/6 | |
| Liver | Inflammatory cell infiltration, peribiliary (intrahepatic) | (+1) | 0/16 | 0/5 | 2/5 | 5/6 | 2/6 | |
|  |  | (+2) | 0/16 | 0/5 | 0/5 | 0/6 | 2/6 | |
|  |  | (+3) | 0/16 | 0/5 | 0/5 | 0/6 | 2/6 | |
|  | Bile duct hyperplasia | (+1) | 0/16 | 0/5 | 3/5 | 4/6 | 4/6 | |
|  |  | (+2) | 0/16 | 0/5 | 0/5 | 1/6 | 2/6 | |

*Severity grade: (1+) = mild, (2+) = moderate, (3+) = marked
